# Role of the intrinsic subtalar ligaments in subtalar instability and consequences for clinical practice

**DOI:** 10.3389/fbioe.2023.1047134

**Published:** 2023-03-10

**Authors:** Frederick Michels, Evie Vereecke, Giovanni Matricali

**Affiliations:** ^1^ Orthopaedic Department AZ Groeninge, Kortrijk, Belgium; ^2^ MIFAS by GRECMIP (Minimally Invasive Foot and Ankle Society), Merignac, France; ^3^ ESSKA-AFAS Ankle Instability Group, Kortrijk, Belgium; ^4^ Institute for Orthopaedic Research and Training (IORT), Department of Development and Regeneration, Faculty of Medicine, KU Leuven, Leuven, Belgium; ^5^ EFAS European Foot and Ankle Society, Brussels, Belgium; ^6^ Department Development and Regeneration, Faculty of Medicine, University of Leuven Campus Kortrijk, Kortrijk, Belgium; ^7^ Department of Orthopaedics, Foot and Ankle Unit, University Hospitals Leuven, KU Leuven, Leuven, Belgium

**Keywords:** subtalar instability, cervical ligament, ankle sprain, anatomy, pathophysiology, calcaneofibular ligament, talofibular ligament, interosseous talocalcaneal ligament

## Abstract

Subtalar instability (STI) is a disabling complication after an acute lateral ankle sprain and remains a challenging problem. The pathophysiology is difficult to understand. Especially the relative contribution of the intrinsic subtalar ligaments in the stability of the subtalar joint is still controversial. Diagnosis is difficult because of the overlapping clinical signs with talocrural instability and the absence of a reliable diagnostic reference test. This often results in misdiagnosis and inappropriate treatment. Recent research offers new insights in the pathophysiology of subtalar instability and the importance of the intrinsic subtalar ligaments. Recent publications clarify the local anatomical and biomechanical characteristics of the subtalar ligaments. The cervical ligament and interosseous talocalcaneal ligament seem to play an important function in the normal kinematics and stability of the subtalar joint. In addition to the calcaneofibular ligament (CFL), these ligaments seem to have an important role in the pathomechanics of subtalar instability (STI). These new insights have an impact on the approach to STI in clinical practice. Diagnosis of STI can be performed be performed by a step-by-step approach to raise the suspicion to STI. This approach consists of clinical signs, abnormalities of the subtalar ligaments on MRI and intraoperative evaluation. Surgical treatment should address all the aspects of the instability and focus on a restoration of the normal anatomical and biomechanical properties. Besides a low threshold to reconstruct the CFL, a reconstruction of the subtalar ligaments should be considered in complex cases of instability. The purpose of this review is to provide a comprehensive update of the current literature focused on the contribution of the different ligaments in the stability of the subtalar joint. This review aims to introduce the more recent findings in the earlier hypotheses on normal kinesiology, pathophysiology and relation with talocrural instability. The consequences of this improved understanding of pathophysiology on patient identification, treatment and future research are described.

## 1 Introduction

Lateral ankle sprains are the most prevalent musculoskeletal injuries in the physically active population and form an important health problem given the associated high socio-economic cost (Paterson et al., 2000). Moreover, a significant part of these patients develops chronic complaints of hindfoot instability (Michels et al., 2021a). These symptoms are often related to mechanical impairments at the joints of the hindfoot which result in different forms of instability such as talocrural, subtalar, syndesmosis, medial and Chopart instability (Hertel and Corbett, 2019; Bleakley et al., 2021). The most known type of instability is lateral talocrural instability (Usuelli et al., 2014). The different aspects related to the pathophysiology of lateral talocural instability have been well described in literature and the most commonly involved ligaments, the anterior talofibular ligament (ATFL) and calcaneofibular ligament (CFL), are well known (Matsui et al., 2017a; Rochelle et al., 2020). Besides the pathophysiology, there is agreement on diagnosis and treatment (Michels et al., 2018).

In contrast to talocrural instability, the literature about subtalar instability (STI) is far more limited and until recently its pathophysiology remained less well known. The contribution of the subtalar ligament in the stability of the subtalar joint is a main research question. The limited understanding often results in misdiagnosis and inappropriate treatment (Barg et al., 2012; Aynardi et al., 2015). STI can be defined as a clinical condition with symptoms of instability related to a pathological laxity at the level of the subtalar joint. An involvement of the subtalar joint has been estimated in about 25% of all the cases presenting with talocrural instability (Larsen, 1988; Tourné et al., 2010; Guillo et al., 2013; Aynardi et al., 2015; Mittlmeier and Rammelt, 2018).

STI is a challenging problem for different reasons. First, the literature about the anatomy of the subtalar ligaments is equivocal (Jotoku et al., 2006; Poonja et al., 2017; Mittlmeier and Rammelt, 2018). In literature addressing subtalar instability, there is some confusion about the terminology because different authors use different terms for the same structure and similar terms for different structures. Moreover, literature about their dimensions and precise anatomical location is limited. Second, the pathophysiology of subtalar instability is still difficult to understand and subject to debate (Mittlmeier and Rammelt, 2018). The relative contribution of each ligamentous structure in the stability of the subtalar joint is unclear. While most researchers agree that the CFL is an important stabilizer of both the tibiotalar and the subtalar joint (Kjaersgaard-Andersen et al., 1987; Li et al., 2019), some consider the interosseous talocalcaneal ligament (ITCL) as the second most important stabilizer (Kjaersgaard-Andersen et al., 1987; Sarrafian S. K., 1993; Pisani, 1996; Knudson et al., 1997). Others recognize the cervical ligament (CL) as an important stabilizer of the subtalar joint (Meyer et al., 1988; Kato, 1995) whereas this ligament is neglected in some biomechanical studies assessing STI (Heilman et al., 1990; Ishii et al., 1996; Weindel et al., 2010; Krähenbühl et al., 2019). Third, the group of patients with chronic hindfoot instability complaints is not homogeneous and patients with STI are difficult to discern from those with talocrural and other instabilities (Aynardi et al., 2015; Mittlmeier and Rammelt, 2018). This is largely due to the fact that the joints in this region are connected anatomically and functionally. They are stabilised by several ligaments that are prone to both isolated and combined ligamentous injuries (Bleakley et al., 2021). This close connection results in symptoms and clinical signs of STI that are overlapping with those of talocrural instability and other instabilities (Aynardi et al., 2015; Mittlmeier and Rammelt, 2018).

Finally, the reliability of the different diagnostic tools is unclear (Tourné and Mabit, 2017). Stress radiographs are commonly used for diagnosis but several publications question the reliability of those methods. Other diagnostic tests have been published but are not commonly used.

The difficulties in diagnosis and treatment result in persisting complaints of instability despite a surgical treatment (Broström, 1965; Barg et al., 2012; Michels et al., 2021b). Therefore, an improved understanding of the different aspects related to STI is helpful to improve the approach to patients with STI.

The purpose of this review is to provide a comprehensive update of the current literature related to the pathophysiology of STI. The hypothesis of this review is that the intrinsic subtalar ligaments play an important role in the stability of the subtalar joint. Recent literature clarifies the local anatomical and biomechanical characteristics of the subtalar ligaments. This allows to improve the understanding of kinesiology and pathophysiology (Michels et al., 2020a; Michels et al., 2021b; Pereira et al., 2021). This improved understanding has some consequences for clinical practice.

A review of the literature was performed to identify peer-reviewed articles about 1) the pathophysiology, diagnosis and treatment of STI and 2) anatomical and biomechanical properties of the intrinsic subtalar ligaments. A comprehensive search in the following databases was performed: PubMed, Web of Science and, Embase. This review aimed to introduce the more recent findings in the earlier hypotheses on normal kinesiology, pathophysiology and relation with talocrural instability. The consequences of this improved understanding of the pathophysiology on patient identification, treatment and future research are described.

## 2 Update on the anatomical and biomechanical aspects of the subtalar joint

The basic mechanical stability of the subtalar joint is determined by a complex interaction between articulating bones and different ligaments.

### 2.1 The bony aspects

The subtalar joint consists of two articulating components: the posterior and the anterior component. The posterior subtalar joint has an ovoid shape and is formed by the relatively concave posterior facet of the talus and the convex posterior facet of the calcaneus (Sarrafian S. K., 1993). The anterior component corresponds to the head of the talus, the middle and anterior facet of the calcaneus and the navicular bone. Together, they are indicated as the coxa pedis. The posterior and anterior component function together and are often referred to as the peritalar complex (Sarrafian S. K., 1993). The variation in bone and joint morphology will affect joint mechanics. Varus malalignment of the hindfoot has been shown to increase the risk of chronic instability (Bonnel et al., 2010; Lintz et al., 2019). A recent study demonstrated an association between a more plantarly and valgus oriented posteroinferior talar facet and complaints of chronic instability (Kleipool et al., 2021). However, the impact of joint morphology on the occurrence of instability complaints remains largely unexplored and is an opportunity for further research.

### 2.2 The soft tissue aspects

A complex of ligamentous structures connects the talus to the calcaneus. We distinguish the extrinsic and intrinsic subtalar ligaments which contribute to joint stability.

The most important extrinsic ligaments are the CFL, which limits inversion, and deltoid ligament, which limits eversion. Some studies indicate that the anterior talofibular ligament (ATFL) has an indirect function in the stability of the subtalar joint (Cass and Settles, 1994; Hollis et al., 1995; Boey et al., 2019).

The talus and calcaneus are directly connected by the intrinsic subtalar ligaments. A recent publication clarified the confusing terminology that has been used in the past (Table 1). (Jotoku et al., 2006; Poonja et al., 2017; Mittlmeier and Rammelt, 2018; Michels et al., 2021c) We distinguish the CL, ITCL and the anterior capsular ligament (ACaL). Varying concepts on the relative contribution of these ligaments have been published. Today, several recent publications support the hypothesis that the intrinsic subtalar ligaments play an important role in the stability of the subtalar joint. Recent anatomical research clarified the local anatomy and demonstrated that the intrinsic subtalar ligaments have a consistent presence, location and morphology (Jotoku et al., 2006; Poonja et al., 2017; Michels et al., 2022a). Another study tested the hypothesis that these structures have different material properties. The findings in these studies clarify some uncertainties about the ligament function that may yield new insights into the pathomechanics of STI. To this end we provide a summary of the anatomical configuration of these ligaments based on recent literature.

**TABLE 1:** Terminology used to indicate the subtalar ligaments.

Most commonly used term	Other terms
Cervical ligament	• Ligament of Fick Viladot et al. (1984)
	• Anterolateral talocalcaneal ligament Shellshear and Macintosh (1949); Mabit et al. (1997)
	• Anterior talocalcaneal ligament Testut and Latarjet (1985); Mabit et al. (1997)
	• Oblique talocalcaneal ligament Mittlmeier and Rammelt (2018)
	• External talocalcaneal ligament Smith (1896)
	• Lateral band of the interosseous ligament Last (1952)
	• Cervical talocalcaneal ligament Mabit et al. (1997)
Interosseous talocalcaneal ligament	• “Hedge” ligament of Farabeuf Mabit et al. (1997)
	• Ligament of the tarsal canal Viladot et al. (1984)
	• Oblique astragalo-calcanean ligament Smith (1896)
	• Cruciate ligament of the tarsus Viladot et al. (1984)
	• Axial ligament Viladot et al. (1984)
Anterior capsular ligament	• Posterior capsular ligament Li et al. (2013b)
	• Ligament of the anterior capsule of the posterior talocalcaneal joint Stephens and Sammarco (1992)
	• Anterior talocalcaneal ligament Mittlmeier and Rammelt (2018)

#### 2.2.1 Anterior talofibular ligament

The ATFL commonly consists of two bands: the superior and inferior fascicle, which are characterized by distinct anatomical and biomechanical properties (Vega et al., 2020). Both fascicles have a confluent origin on the anterior distal border of the fibula. The insertion of the superior fascicle corresponds to the body-neck junction of the talus while the insertion of the inferior bundle is located more plantarly on the talar body. The superior fascicle becomes lax in dorsiflexion, and taut in plantarflexion. The inferior fascicle is a more isometric structure and has a confluent origin with the CFL (Vega et al., 2020). Studies on the material properties of the ATFL demonstrate a low failure load and a low stiffness (Siegler et al., 1988; Waldrop et al., 2012; Rochelle et al., 2020). The low failure load may explain the high occurrence of ATFL injuries.

#### 2.2.2 Calcaneofibular ligament

The CFL is bridging both the talocrural and subtalar joint. In a recent study, Pereira et al. described four distinct morphological-oriented shapes of the CFL: 1) a single bundle (44.7%), 2) Y-shape double bundle, 3) V-shape double bundle, and 4) a separated bundle (Pereira et al., 2020). Type 4 is uncommon and corresponds to the presence of an associated lateral talocalcaneal ligament. In the single bundle type there is no direct connection between talus and calcaneus while there is in the other types. It remains to be investigated if there is a relation between the morphological variants of the CFL and development of STI.

Studies assessing the material properties of both the CFL and ATFL demonstrated a higher failure load of the CFL compared to that of the ATFL, supporting its important role in stabilizing the subtalar joint (Attarian et al., 1985; Siegler et al., 1988; Nigg et al., 1990; Rochelle et al., 2020). The CFL gets tensioned in inversion and dorsiflexion, and will stabilize the joint in these positions, while it is lax in eversion and plantarflexion (Colville et al., 1990; Cawley and France, 1991; Bahr et al., 1998; Ozeki et al., 2002). The inferior fascicle of the ATFL and CFL are connected with some arciform fibers that allow some transmission of tension (Cordier et al., 2021), and together they are referred to as the lateral fibulotalocalcaneal ligament complex of the ankle (Vega et al., 2020).

#### 2.2.3 The cervical ligament

**FIGURE 1:**
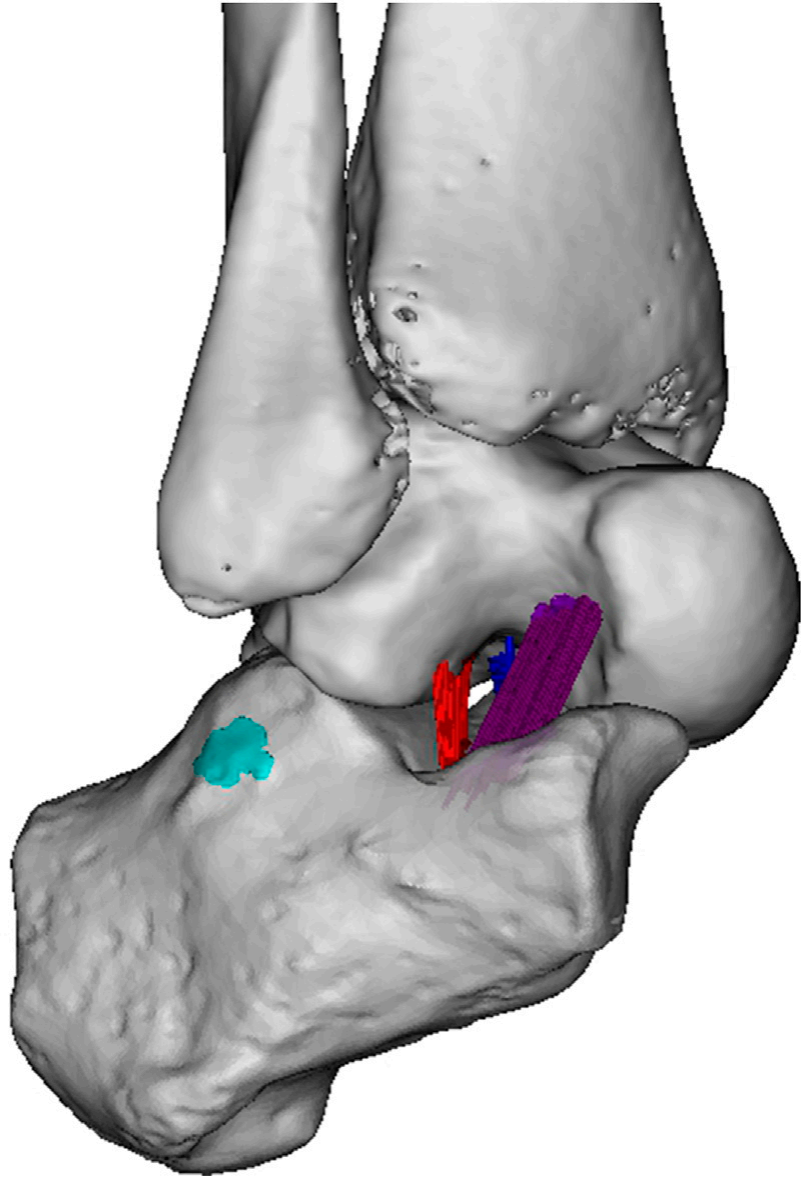
Subtalar ligaments. Sinus tarsi view. The following structures are indicated in colour: ITCL fibers (dark blue), CL fibers (purple), ACaL fibers (red) and CFL footprint (cyan blue). (Figure 2G in Foot and Ankle Surg 2021 January; 27 (1):101–109, no permission needed).

The cervical ligament is located in the sinus tarsi and has similar dimensions as the ATFL and CFL (Michels et al., 2021c). In neutral position, the CL runs from the antero-superior and medial side of the talus to the posteroinferior and lateral side of the calcaneus (Figure 1). In inversion, the CL is taut in a vertical position, while in eversion the CL is taut in a horizontal position. Anterior drawer of the calcaneus in neutral position also tensions the CL. This is in contrast to the CFL which is not tensioned during anterior drawer and everted position.

The CL has a low failure load and a low stiffness, similar to the ATFL (Michels et al., 2022a). The CL is more compliant than the ITCL, which may beneficial in the adaptation of the foot to the ground, while the low failure load may result in a higher susceptibility to injuries.

#### 2.2.4 The interosseous talocalcaneal ligament

The ITCL is a vertical ligament located in the tarsal canal. Both attachment sites or footprints of the ligament have a linear shape that run parallel with the tarsal canal (Michels et al., 2021c). The ITCL is significantly thinner and shorter than the ATFL, CFL and CL. Several morphological variations of the ITCL were observed: band type, fan type, and multiple type (Jotoku et al., 2006; Michels et al., 2020b). The ACaL-ITCL complex has a rather high failure load and high stiffness (Michels et al., 2022a). This supports its role in maintaining the apposition of the subtalar joint and allowing inversion and eversion (Cahill, 1965; Pisani, 1996; Tochigi et al., 2004; Michels et al., 2022a).

#### 2.2.5 The anterior capsular ligament

The ACaL is a vertical rectangle band and corresponds to a thickened part of the anterior joint capsule of the posterior facet (Sarrafian S., 1993; Michels et al., 2021c). The ACaL is often seen as the ITCL or as a second band of the ACaL. Publications that are not clear about this should be interpreted with caution. The footprints of the ACaL are mainly located in the sinus tarsi but often continue inside the tarsal canal. The ACaL and ITCL, taken together, are called the ‘hedge’ ligament of Farabeuf as they are wide and short and run in the same direction (Mabit et al., 1997). The ACaL can be easily seen from a lateral view into the sinus tarsi and from an intra-articular point of view (Michels et al., 2021c).

## 3 Kinematics of the subtalar joint

The kinematics of the subtalar joint correspond to the major functions of foot: support and propulsion. The foot acts as a flexible structure to adapt to uneven surfaces but also as rigid structure to allow force transmission. The subtalar joint plays a key role in both functions and the transition between both functions. The unique triplanar motion of the subtalar joint is a direct result of the interaction between the bony shape of the articular surfaces of the PTC and TCN joints and the ligaments (Sarrafian S. K., 1993). As the talus has no muscle attachments, the subtalar joint acts as a passive flexible structure. Motion in this joint is indirectly influenced by external forces, such as muscle and ground reaction forces. Different concepts have been presented to describe the kinematics of the subtalar joint.

### 3.1 Uniaxial motion concept

In a simplified concept, motion at the subtalar joint can be seen as uniaxial motion resulting in eversion and inversion. The motion is triplanar: eversion is intrinsically linked to dorsiflexion and abduction, whereas inversion is linked to plantar flexion and adduction of the foot. From eversion to inversion the calcaneus rotates with respect to the talus from dorsolateral to medioplantar. In eversion, maximum contact caused by joint surface congruency at the posterior subtalar joint generates a high degree of inherent stability (Sarrafian S. K., 1993). In inversion, congruency decreases resulting in a more important role for the ligaments in keeping the stability. The decreased congruency also results in higher contact pressures in this inverted position (Wagner et al., 1992).

**FIGURE 2:**
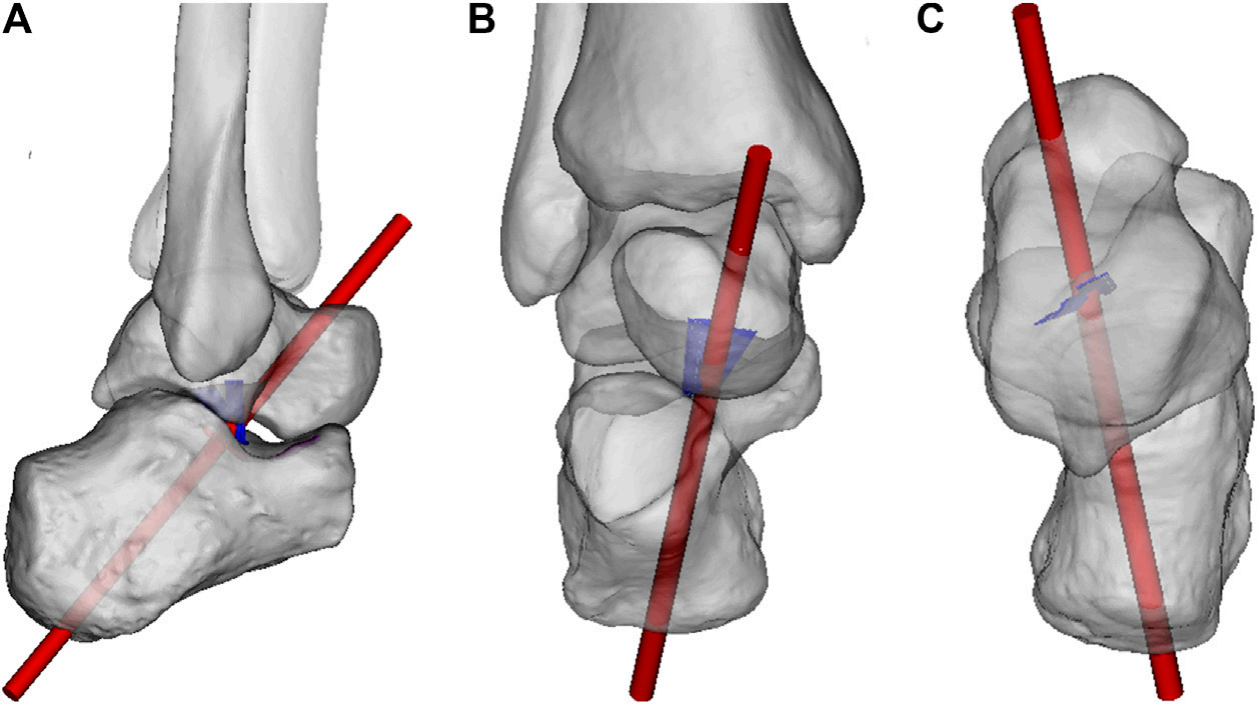
Axis of rotation of the subtalar joint. **(A)**. Lateral view **(B)**. Anterior view **(C)**. Superior view. ITCL fibers indicated in blue.

**FIGURE 3:**
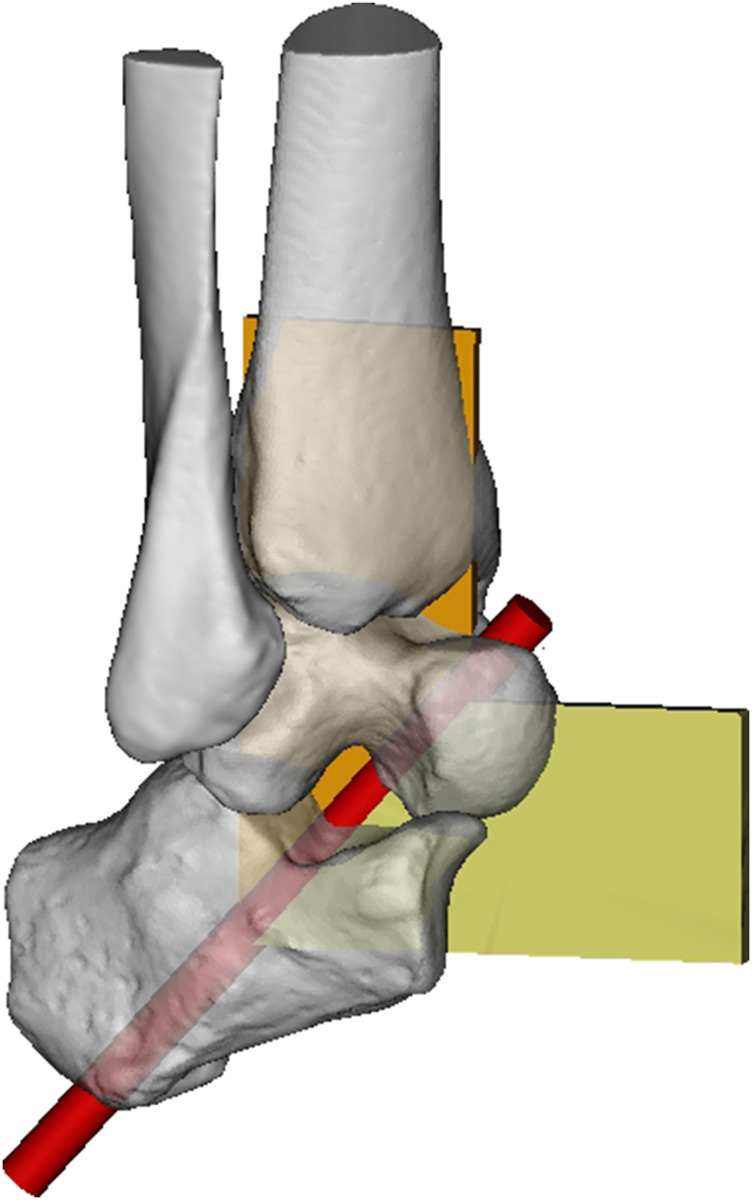
Concept of the subtalar joint as mitred hinge.

The uniaxial motion can be described by a single oblique axis of motion that runs obliquely from posteroplantolateral to anterodorsomedial (Goto et al., 2009; Peña Fernández et al., 2020). It penetrates the midpoint of the heel and the talar neck (Figure 2). A recent study assessed the centre of rotation of the subtalar joint during inversion and eversion motion using full weight-bearing clinical computed tomography (Peña Fernández et al., 2020). It demonstrated that this centre of rotation is located in the middle facet of the subtalar joint. An MRI study assessing the 3D *in vivo* kinematics demonstrated that the footprints of the ITCL are very close to the axis of rotation (Goto et al., 2009). The short ITCL has a relatively high stiffness and high failure load compared to the cervical ligament. These recent findings confirm the earlier concept that the subtalar joint can be seen as a mitred hinge with the ITCL as central pivot (Figure 3).

### 3.2 Multiaxial motion concept

Although this basic uniaxial motion concept is well accepted and useful in clinical practice, it should be seen as a simplification. A more complex concept depicts a combination of sliding, rolling and spinning at the joints with the ITCL and CL as most important guiding ligaments (Sarrafian S. K., 1993). Both ligaments remain tight during inversion and eversion (Sarrafian S. K., 1993). The articular surfaces of the posterior and anterior component have a complex configuration but may be classified as male and female ovoid surfaces (Sarrafian S. K., 1993). The mobility is restricted by the tight ligaments resulting in a complex twisting motion. A motion of the anterior component can only occur as long as the posterior component accommodates in an opposite direction.

**FIGURE 4:**
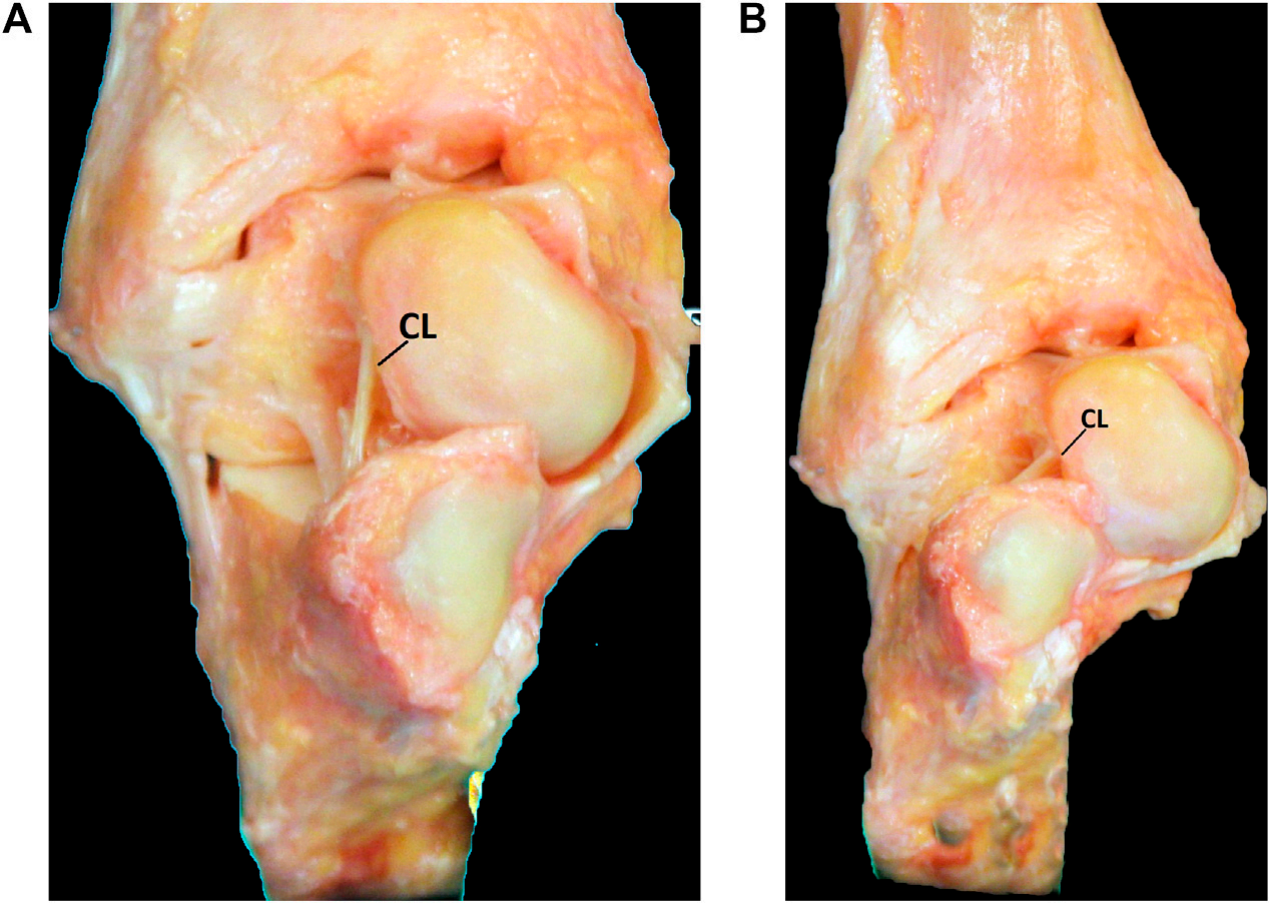
Anterior view of the hindfoot. The CL allows a swinging motion of the calcaneus around the talar neck. **(A)**. In inversion, the CL is taut in a vertical position. **(B)**. In eversion the CL is taut in a more horizontal position.

Recent findings about the CL clarify its function in this multiaxial motion concept. As the CL is located distantly from the centre of movement, it allows a swinging motion of the calcaneus around the talar neck (Figure 4). The CL has a relatively low failure load, with values that correspond with the failure load of the ATFL (St Pierre et al., 1983; Attarian et al., 1985; Rochelle et al., 2020). The lower load to failure of the CL, comparable to the ATFL, places the CL at risk for rupture during the same inversion motion, causing an ankle sprain.

As the ACaL appears to have different anatomical characteristics, it should no longer be confused with the ITCL. The ACaL plays a rather supporting role in this multiaxial motion. The laterally located ACaL fibres are longer and have more excursion than the medially located ITCL fibres (Sarrafian S. K., 1993; Stagni et al., 2003). This explains the inversion related to the anterior translation of the calcaneus. The ACaL is taut with inversion and loose with eversion. The footprint of the ACaL is oriented perpendicularly to the axis of motion, which makes the lateral part more vulnerable to injury. This explains the findings in MRI studies reporting a correlation between ACaL lesions and STI (Kim et al., 2017; Yoon et al., 2018; Jung et al., 2021). Besides inversion and eversion, both ITCL and ACaL get tensioned with anterior and posterior translation of the calcaneus and may provide stability in the anteroposterior direction. Next to the intrinsic ligaments, the extrinsic ligaments such as the deltoid ligament and CFL ligament provide further support by limiting eversion and inversion respectively.

## 4 Update on pathophysiology of STI

Several hypotheses about the injury mechanisms related to STI have been described but are not yet confirmed (Lee et al., 2016). An inversion movement may be the most probable mechanism. Video analysis demonstrated that inversion is the primary injury mechanism by which lateral ankle sprains occur (Fong et al., 2012). Inversion is also the mechanism that produces medial subtalar dislocation. Somewhere in between is an injury that results in instability of the subtalar joint as an isolated problem or as a component of other injuries (Clanton, 1989).

Meyer et al. proposed two different mechanisms that involve the subtalar ligaments (Meyer et al., 1988). The first injury mechanism corresponds to a hindfoot supination occurring during ankle plantarflexion. This would result in the following order of ligament rupture: ATFL, CFL, lateral capsule and finally the ITCL. The second injury mechanism corresponds to a supination force on an ankle in dorsiflexion. This would result in the following order of ligament injury: CFL, CL and finally the ITCL. Another type of injury leading to STI that has been described is a whiplash mechanism caused by an abrupt impact and deceleration of the calcaneus resulting in an injury of the ITCL and CL (Pisani, 1996).

The pathological process starts with a first ankle injury resulting in one or more damaged ligaments. As there is a narrow interdependence between the talocrural and subtalar joints, lesions may occur at both levels and will have an impact on each other. After the injury, healing processes and coping mechanisms will affect the further outcome (Hertel and Corbett, 2019). However, the primary lesions may also result in disturbed kinematics, increased stress and secondary injuries. This diversity of lesions and consequences leads to different injury patterns and different concepts.

### 4.1 The concept of micro-instability

An ATFL lesion is the most commonly injured ligament during an ankle sprain. An isolated ATFL lesions occurs in 35%–65% of the lateral ankle sprains (Broström, 1965; van den Hoogenband et al., 1984). Kemmochi et al. demonstrated a high rate of incomplete ATFL lesions (Kemmochi et al., 2016). Plantarflexion-supination is the most likely injury mechanism. The concept of micro-instability corresponds to a lesion of the superior fascicle of the ATFL (Vega et al., 2020). This partial ATFL lesion can cause remaining symptoms but the long-term consequences are unknown. Currently, there is no agreement about the need for early surgical treatment as it seems unlikely that an incomplete lesion of the ATFL significantly increases the instability of the subtalar joint.

### 4.2 The rotational concept

A complete lesion of the ATFL is not uncommon. This involves the interruption of both fascicles of the ATFL. A rupture of the ATFL allows the talus to move forward to the fibula resulting in an instability of the talocrural joint. In clinical examination this corresponds to a positive anterior drawer test.

Although the ATFL does not bridge the subtalar joint, several biomechanical studies also demonstrate a relation between an isolated sectioning of the ATFL and abnormally increased mobility of the subtalar joint (Cass and Settles, 1994; Hollis et al., 1995; Boey et al., 2019). Cass et al. studied the kinematics of ankle instability after lateral ligament sectioning in a model where axial rotation was not constrained (Cass and Settles, 1994). External rotation of the tibiofibular complex increased after sectioning of the ATFL resulting in an increased inversion. They found no tilting of the talus in the mortise to occur with isolated release of the ATFL or CFL. Hollis et al. performed a sectioning of the ATFL and demonstrated an increased dorsiflexion in the talocrural joint but also an increased motion in the subtalar joint (Hollis et al., 1995). The greatest increase for subtalar joint motion was rotation, which was especially seen in dorsiflexion and less in neutral position. Boey et al. demonstrated an increased range of motion in internal-external rotation in the subtalar joint but a decreased ROM in dorsiflexion-plantarflexion in the tibiotalar joint (Boey et al., 2019).

**FIGURE 5:**
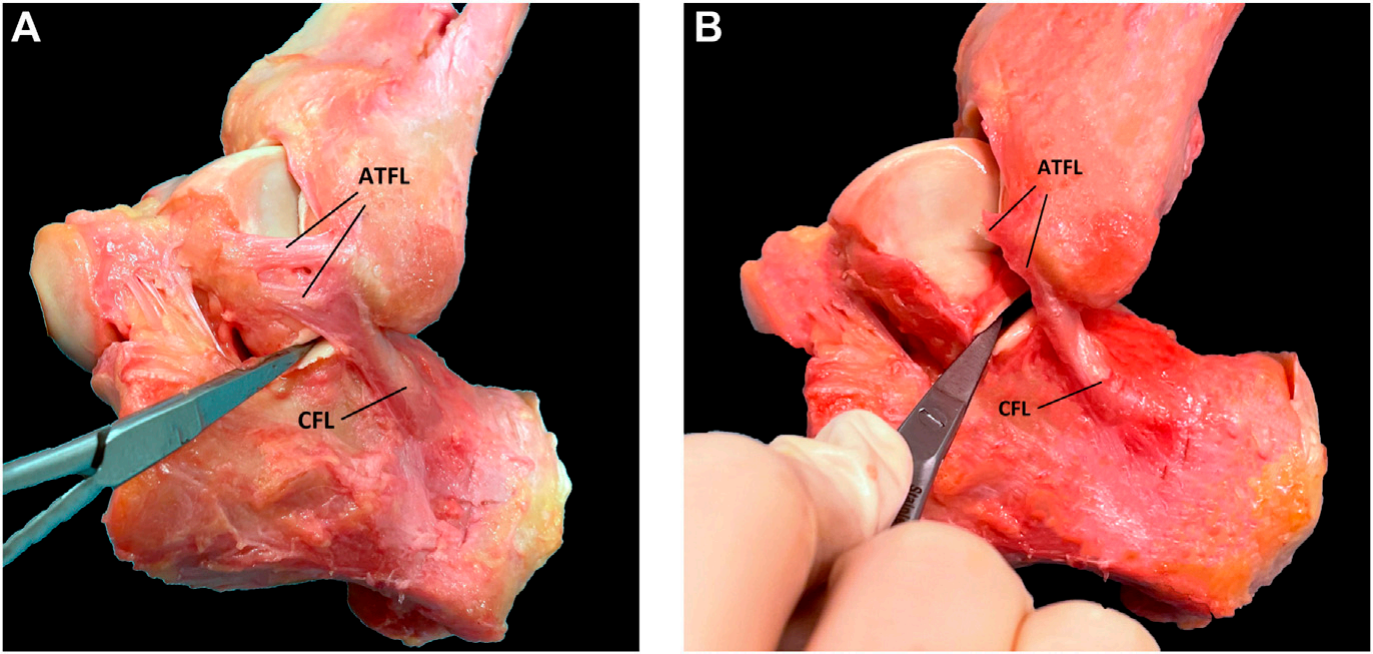
The rotational concept. **(A)**. Lateral view on the subtalar joint before sectioning the ATFL **(B)**. After sectioning the two bundles of the ATFL the talus moves in an anterior direction and the tension of the CFL decreases. This allows increased opening of the posterior facet with intact subtalar ligaments and intact CFL.

These findings can be explained by a complex interaction of the ATFL and CFL. Indirectly, the intact ATFL holds the CFL under tension. An intact ATFL limits the anterior translation of the talus to the fibula and thereby retains the fibula its normal position. In a more direct way, the arciform fibers may transmit tension from the intact ATFL to the CFL (Cordier et al., 2021). This transmission of tension is more important with the talocrural joint in dorsiflexion which secures the joint and tensions the CFL. In case of a complete interruption of the ATFL, the talus rotates internally allowing the fibula to move toward the calcaneus and thereby making the CFL lax. The loss of anterior talofibular restraint, unlocks the subtalar joint, allowing further inversion (Figure 5). (Hintermann, 1999) The increase of inversion may result in symptoms of instability without tilting at the talocrural joint. This complex interaction explains why symptoms of ankle instability can occur in the absence of radiographically demonstrable talar tilt. The same concept corresponds with MRI findings of an enlarged angle between ATFL and posterior tibiofibular ligament after rupture of the ATFL (Li et al., 2020a). This concept also explains the relation between an ATFL lesion and a disturbed dorsiflexion causing an anterior impingement on the long term.

It remains unknown what the long term impact is of such isolated ligament lesion on the other ligaments. The altered kinematics may cause further ligament damage and result in a multiligament involvement.

### 4.3 Isolated CFL lesions and combined ATFL and CFL lesions

An isolated injury of the CFL seems to be very rare in clinical practice (Francillon, 1962; Rigby et al., 2015; Mittlmeier and Rammelt, 2018). The limited reports in literature only described the intact ATFL and the injured CFL, they did not assess the subtalar ligaments.’

Despite the low occurrence of isolated CFL lesions, many biomechanical studies demonstrated that an isolated sectioning the CFL has an important impact on the stability of the subtalar joint (Kjaersgaard-Andersen et al., 1987; Heilman et al., 1990; Stephens and Sammarco, 1992; Cass and Settles, 1994; Martin et al., 1998; Martin et al., 2002). These studies support the recommendation to repair or reconstruct the CFL in case of injury as a part of a more extensive technique treating multiple ligament lesions.

A combined ATFL and CFL injury is the second most commonly reported injury pattern to the lateral ankle ligament complex. A recent MRI study with 180 patients reports ATFL lesions in 96.3% of all 108 included patients with CFL lesions (Debieux et al., 2020). The occurrence of combined ATFL and CFL lesions varies from 20% to 65% (Broström, 1965; van den Hoogenband et al., 1984). A biomechanical study demonstrated that sectioning both ligaments caused a positive talar tilt in all specimens corresponding to a major instability (Cass and Settles, 1994). There is, however, a lack of clinical studies that have investigated if CFL and/or ATFL lesions occur in conjunction with lesions of other subtalar ligaments.

### 4.4 Multiligament concept

Lesions of the intrinsic subtalar ligaments are difficult to identify and are probably underreported. These lesions are most commonly associated with lesions of ATFL and CFL (Tochigi et al., 1998; Lee et al., 2016). They are correlated with increased signs of STI. Tochigi et al. performed MRI in 24 patients after an acute ankle sprain (Tochigi et al., 1998). Lesions of the intrinsic subtalar ligaments were found in 18 of the 24 patients. They found a significant relationship between lesions of the ITCL or CL and instability complaints after 12 months.

Also in a chronic situation, lesions of the intrinsic subtalar ligaments are not uncommon. Tourné et al. found lesions of the CL and/or ITCL in 30% of patients in 150 patients selected for a surgical stabilisation procedure (Tourné et al., 2012). Lee et al. found additional CL insufficiency in 19 out of 66 patients with combined ATFL and CFL lesions (Lee et al., 2016). CL insufficiency was correlated with radiographic signs of STI. Martin et al. demonstrated in two biomechanical studies that a disruption of the CFL results in increased strain of the CL in open and closed kinetic chain (Martin et al., 1998; Martin et al., 2002). Taken into account the lower load to failure of the CL, it becomes more vulnerable to secondary injury in case of CFL lesions. Thus, in a longstanding chronic instability it seems more likely that multiple ligaments are involved subsequently due to altered kinematics and repetitive microtrauma (Mittlmeier and Rammelt, 2018).

### 4.5 Partial lesions

Currently, the relevance of partial lesions is still unknown. Karlsson et al. described their results in 176 patients with chronic symptoms of instability (Karlsson et al., 1988). In all their patients, the ligaments were found to be scarred and elongated, but in continuity. A recent biomechanical study tested the ligaments during uniaxial loading and demonstrated a high occurrence of partial ligaments ruptures (Michels et al., 2022a). A partial rupture may result in a ligament losing its property to resist tensile forces while the anatomical continuity remains. Anatomical continuity may be important to guide the healing process. It is not yet know if this healing process results in a full restoration of the ligament properties. A weakening of the ligament may also transfer forces to other ligaments resulting in secondary lesion.

The occurrence of partial lesions is also a diagnostic challenge as they may be difficult to recognize with imaging techniques such as ultrasound and MRI. Also during intraoperative assessment, the visual presence of the ligament may misguide the interpretation of the remaining stabilising capacities. Therefore, partial lesions are more likely to pass unnoticed. The impact of these lesions on joint stability needs further investigation.

## 5 Discussion: Consequences for clinical practice

### 5.1 Consequences for patient identification

**FIGURE 6:**
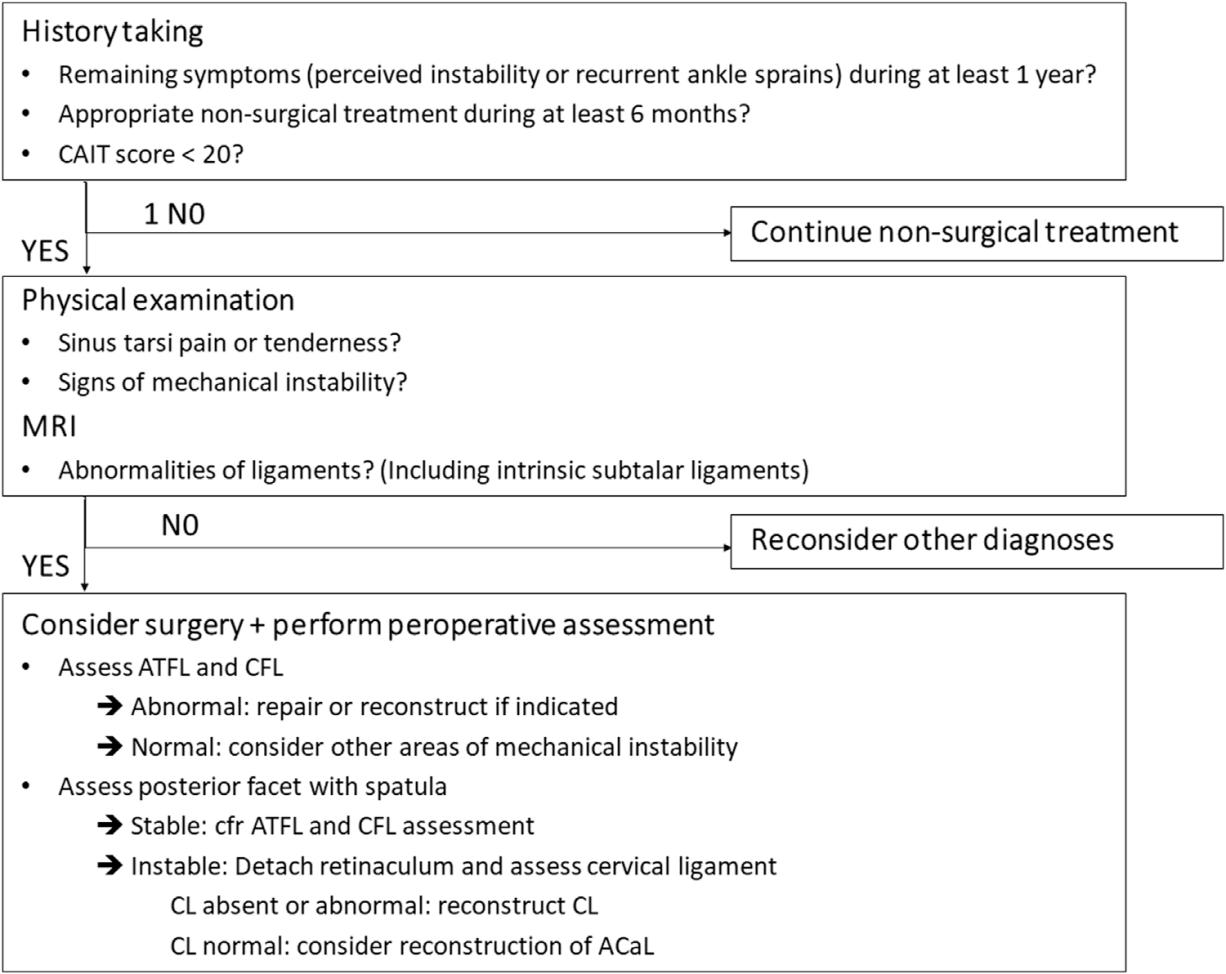
Algorithm (Figure 1 in Foot and Ankle Surg 2022 June. 10.1016/j.fas. 2022.06.006, no permission needed).

Diagnosis of STI is still challenging but several clinical and radiographic signs seem to be correlated with a subtalar origin of the complaints. The earlier discussed new insights allow to improve interpretation of injury patterns during imaging and also during surgery. Recently published research used these different aspects to present an algorithm to diagnose and treat STI in clinical practice (Figure 6). (Michels et al., 2022b) A step-by-step approach is recommended to improve patient identification and decision taking.

The first step, during history taking, is considering this diagnosis in case of persistent symptoms of instability. Recent research demonstrated a correlation between persistent symptoms and abnormalities at the subtalar joint (Michels et al., 2020a). Subjective instability, recurrent ankle sprains and giving way are the most common symptoms related to instability. Patients with STI usually have a history of acute ankle sprain. They may complain of lateral hindfoot pain and a recurrent feeling of instability of the ankle like ‘rolling over’ or ‘giving way’ (Keefe and Haddad, 2002). The symptoms are worse when performing sports activities or walking on uneven ground (Karlsson et al., 1998; Aynardi et al., 2015).

The following step corresponds to physical examination. Painful areas may indicate the most plausible origin of the complaints. Tenderness in the sinus tarsi is a typical symptom that may indicate involvement of the intrinsic subtalar ligaments. Lateral ankle swelling and stiffness may be present. An increase in inversion on of the subtalar joint and an increase in forward translation of the calcaneus beneath the talus may be found (Thermann et al., 1997; Keefe and Haddad, 2002). There are also some clinical tests described. Thermann et al. tested the chronic anterolateral rotatory instability of the subtalar joint (Thermann et al., 1997). An inversion is applied to the heel while holding the foot in a dorsiflexed position. In case of STI an inversion and internal rotation of the foot can be performed. A medial shift (>5 mm) of the calcaneus under the talus and an opening of the talocalcaneal angle (>5°) correspond to a positive result. Hertel described the medial subtalar glide test (Hertel et al., 1999). The examiner performs a supination and pronation until the talar head is felt equally medially and laterally. While holding the talus in this subtalar neutral position, the calcaneus is glided medially until the talar head could not be palpated or was felt equally medially and laterally.

**FIGURE 7:**
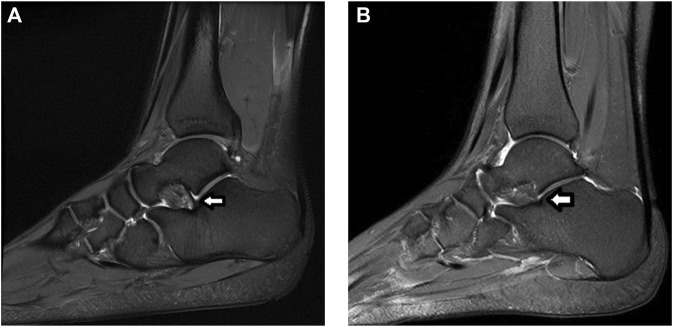
MRI assessment of the ACaL (white arrow) on sagittal image. Proton density image with fat suppression. **(A)**. Normal ACaL. **(B)**. Abnormal ACaL.

In a next step, imaging techniques can be used to challenge this tentative diagnosis. A recent systematic review assessed the different diagnostic tools and found insufficient evidence to use Brodén stress radiographs (Michels et al., 2020a). Anterior drawer-supination radiographs seem to be more reliable but are limited by their two-dimensional assessment and difficult practical implementation (Lee et al., 2016). MRI seems to be a promising diagnostic tool in the assessment of STI (Michels et al., 2020a). MRI offers the advantage of assessing bone and ligaments in three dimensions, even more it allows the assessment of associated injuries (Tourné et al., 2010). Several publications already described the use of MRI to assess the integrity of the ATFL and CFL (Joshy et al., 2010; Xu et al., 2021). The injured ligaments may appear disrupted, thickened, heterogeneous, abnormally contoured or attenuated in signal intensity (Perrich et al., 2009). As the CFL is very difficult to assess on standard views, oblique coronal views should be included in the standard MRI protocol assessing CAI. MRI seems also very valuable in the assessment of the subtalar ligaments. Coronal and coronal oblique planes were found the most useful to assess the inferior extensor retinaculum, CL and ITCL (Mabit et al., 1997). Lee et al. found a correlation between abnormalities of the CL on MRI and complaints of instability (Lee et al., 2016). On MRI, the ACaL can be visualised on sagittal and coronal proton density images (Figure 7). (Linklater et al., 2009) In some cases bone oedema on the footprints or avulsion fractures may be visible. Further research with advanced MRI techniques may allow improvement in the recognition of the different structures. Along with clinical studies this will allow us to correlate the ligament characteristics on MRI with the clinical situation and intraoperative findings.

In a next step, intraoperative evaluation may be helpful to identify subtalar pathology. If surgery seems necessary, one can use this opportunity to add a intraoperative assessment to the earlier findings. The direct view during surgery allows a direct visual assessment of the ligaments and may detect an abnormal opening of the joints during certain movements. Some earlier studies suggested an endoscopic approach to assess the characteristics of the ACaL (Frey et al., 1999; Mittlmeier and Rammelt, 2018; Jung et al., 2021). Abnormalities of this ligament are correlated with STI. Besides the ACaL, the involvement of the CL should also be considered. As the CL is located behind the extensor retinaculum, an open approach may be more appropriate to assess the CL (Michels et al., 2022b). A recent clinical study combined this ligament assessment with an assessment of the stability of the posterior facet (Michels et al., 2022b). Opening of this posterior facet was assessed during a supination movement. The insertion and possibility of twisting a small spatula was seen as a sign of instability. This intraoperative assessment appeared to be very valuable for decision making during the surgical procedure and allowed to address all aspects of the instability.

### 5.2 Consequences for patient treatment

In literature there is already agreement for non-surgical treatment, which is similar to patients with talocrural instability (Aynardi et al., 2015). If the complaints persist despite a prolonged conservative treatment, a surgical treatment can be considered. Different surgical techniques to address hindfoot instability have been described: tenodesis, ligament repair and ligament reconstruction. Below we describe the advantages and drawbacks of the different techniques and their impact in case of STI.

#### 5.2.1 Tenodesis

In earlier literature, the non-anatomical tenodesis techniques were popular. By attaching the proximal part of the peroneus brevis tendon to one or more bones around the ankle joint, the mobility of the different joints becomes limited. As these tenodesis techniques bridge both the talocrural and subtalar joint, they limit the mobility of both the talocrural and subtalar joint. Moreover, they also span and limit the midfoot joints. By limiting the range of motion they are successful in treating related symptoms of instability including subtalar and midfoot instability. However, the non-anatomical approach results in disturbed joint biomechanics, restricted range of motion, inferior functional results and increased risk of degenerative changes of time (Mabit et al., 1998; Krips et al., 2000; Krips et al., 2001; Schepers et al., 2011; Guillo et al., 2013; Vuurberg et al., 2018). Therefore, the use of these non-anatomical tenodesis techniques is no longer recommended but they remain an option as a salvage technique when other techniques are no longer viable options (Vuurberg et al., 2018).

#### 5.2.2 Ligament repair

After the popularity of tenodesis, ligament repair techniques became more popular, with the Broström procedure as most known example (Broström, 1966a; Jolman et al., 2017). A ligament repair consists of a suturing or reinsertion of the torn ligaments to restore the local anatomy (Broström, 1966b; Guillo et al., 2013). Most commonly, the ligament repair is reinforced by other tissue such as the extensor retinaculum corresponding to the Gould modification (Gould et al., 1980). Because the extensor retinaculum remains attached to the calcaneus, this augmentation has a stabilizing effect on the subtalar joint (Choisne et al., 2017).

These repair techniques are straightforward and easy, offering good-to-excellent postoperative results on the short and medium term. The techniques also present some drawbacks though. First, there are some concerns on return of instability in the longer term (Maffulli et al., 2013; Tourné and Mabit, 2017). Second, many study groups exclude more complicated cases of instability such as patients with major instability, earlier surgery, poor tissue quality, STI, high BMI and high-demand activities (Acevedo and Mangone, 2011; Maffulli et al., 2013; Vuurberg et al., 2018; Li et al., 2020b). Finally, very often there is residual tissue of inferior quality and reinforcement is needed to provide sufficient stability (Tourné and Mabit, 2017).

#### 5.2.3 Ligament reconstruction

More recently, the anatomical ligament reconstruction techniques were described (Takao et al., 2005). These techniques aim to restore the local anatomy by replacing the damaged ligament by local tissues or with autograft or allograft tissue. A gracilis autograft is commonly used to replace the ATFL and CFL (Michels et al., 2016a; Guillo et al., 2016; Cordier et al., 2020). The main advantage of these techniques is that they bring new strong tissue to replace the damaged and weakened ligaments. As they aim to restore the normal kinematics they are seen as superior to the tenodesis techniques (Pereira et al., 2021). The surgical procedure is more demanding than the ligament repair technique but good to excellent results are reported (Cordier et al., 2020).

Because the CFL is recognized as an important stabilizer of both the talocrural and subtalar joint (Kjaersgaard-Andersen et al., 1987; Li et al., 2019), many authors recommend a low threshold to reconstruct the CFL in addition to the ATFL (Karlsson et al., 1997; Mittlmeier and Wichelhaus, 2015; Tourné and Mabit, 2017; Michels et al., 2018). The anatomical reconstruction of the CFL is a technically demanding procedure but recent publications offer helpful guidelines (Michels et al., 2016b; Matsui et al., 2017b; Lopes et al., 2018; Michels et al., 2020c; Michels et al., 2020d).

In more complex cases of STI, reconstruction of both the ATFL and CFL may not be sufficient to restore the subtalar stability. Therefore, several authors have proposed a reconstruction of the subtalar ligaments (Pereira et al., 2021). Some publications described the reconstruction of the interosseous talocalcaneal ligament using a partial Achilles tendon graft (Kato, 1995), the anterior half of the peroneus brevis tendon (Pisani, 1996), a gracilis tendon autograft (Liu et al., 2011) or a suture button (So et al., 2020). More recently, newer techniques with straight bone tunnels have been published (Iglesias-durán et al., 2020; So et al., 2020; Michels et al., 2022b).

**FIGURE 8:**
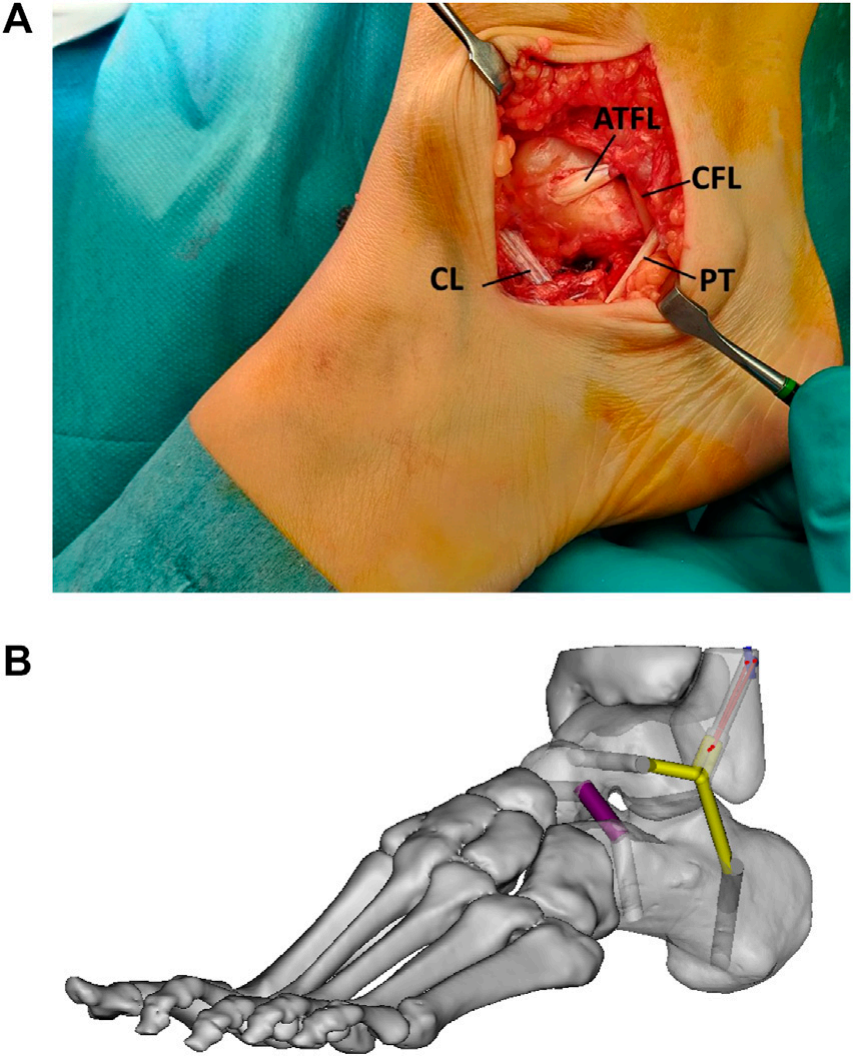
Combined ATFL, CFL and CL reconstruction. **(A)**. Intraoperative superolateral view with reconstructed cervical ligament (CL), reconstructed ATFL (ATFL) and reconstructed CFL (CFL) running behind the peroneal tendons (PT) **(B)**. Image with tunnel positioning. Sinus tarsi view (Figure 4 in Foot and Ankle Surg 2022 June. 10.1016/j.fas. 2022.06.006, no permission needed).

Other publications described the reconstruction of the cervical ligament (Schon et al., 1991; Mabit et al., 1996). Based on recent research, the CL seems the first choice. The CL is important in the normal kinematics and is vulnerable for injuries. Schon et al. published a triligamentous reconstruction attempting to reconstruct ATFL, CFL, and cervical ligament in a near-anatomic fashion (Schon et al., 1991). A plantaris tendon or entire peroneus brevis tendon was used as graft. Mabit et al. described ATFL reconstruction using the peroneus tertius tendon associated with reconstruction of the cervical ligament by inserting a flap of the extensor retinaculum in a talar tunnel (Mabit et al., 1996). A recent clinical study reports good to excellent clinical reports using cervical ligament reconstruction in patients with complex cases with longstanding instability, high BMI, poor tissue quality and generalized hyperlaxity (Michels et al., 2022b). An anatomical reconstruction of the cervical ligament was performed as an isolated procedure or in combination with other procedures according to the specific location of the instability (Figure 8). The reconstruction was performed with newer fixation systems using interference screws in straight bone tunnels. This technical innovation allows a stronger fixation and a more anatomical positioning of the graft (Li et al., 2013a).

### 5.3 Future perspectives

The most recent literature supports the multi-ligament hypothesis to explain the pathophysiology of STI. Further research should focus on the exact injury mechanisms and the exact injury patterns. Such studies should investigate which ligaments are most commonly injured. This should also include the presence of partial lesions as this incidence remains unknown.

In basic research, it would be valuable to develop lab conditions to mimic the *in vivo* condition of an ankle sprain and to assess the different ligaments before and after injury. The injury patterns induced in the lab can be correlated with imaging aspects. The knowledge about the most common injury patterns would help optimize selective sectioning studies that assess the characteristics of chronic instability.

In clinical research, improved imaging techniques may offer a more extensive assessment of acute injuries in clinical practice. Larger observational studies are needed to identify the most common injury patterns and to look for a correlation the primary lesion and long-term symptoms. Studies with repeated MRI may be interesting to look for correlations between chronic and acute conditions. Intraoperative assessment should be compared with imaging and patient related outcome measures.

## 6 Conclusion

Subtalar instability is a challenging complication after an acute lateral ankle sprain. More recent literature focused on this topic resulted in an improved understanding of the pathophysiology of subtalar instability.

Besides the CFL, the intrinsic subtalar ligaments seem to play an important function in the subtalar joint. The CL has a low failure load, similar to the ATFL (Michels et al., 2022a). This low failure load makes it vulnerable to injuries and demonstrates its importance in the pathophysiology of STI.

Injuries of the subtalar ligaments are probably underreported because of their small size and the occurrence of partial ruptures. The different injury patterns are an opportunity for further research.

Diagnosis should be based on a step-by-step approach to raise the suspicion to STI. This approach consists of clinical signs, abnormalities on MRI and intraoperative evaluation. Surgical treatment should address all the anatomical aspects of the instability and focus on a restoration of the normal anatomical situation and its biomechanical properties. A low threshold to reconstruct the CFL is recommended. In complex cases, a reconstruction of the cervical ligament should be considered.
